# Toward energy self-sufficiency in small municipal wastewater treatment plants: assessment of energy recovery potential, process economic balance, and greenhouse gas avoidance

**DOI:** 10.1007/s11356-026-37735-7

**Published:** 2026-04-13

**Authors:** Fernanda Amaral-Góis, Vinicius J. Silva, Renan C. Testolin, Ramaiana Radetski-Silva, Dilamara R. Scharf, Marcelo Poyer-Radetski, Wendell Pimentel-Almeida, Cleder A. Somensi, Rafael Ariente-Neto, Claudemir M. Radetski

**Affiliations:** 1 Instituto Federal Catarinense (IFC), Curso de Mestrado Profissional Em Tecnologia E Ambiente, Campus Araquari, Araquari, SC 89245-000 Brazil; 2 Laboratório de Remediação Ambiental, Universidade Do Vale Do Itajaí (UNIVALI), Itajaí, SC 89302-901 Brazil; 3 Departamento de Química, Universidade Regional de Blumenau (FURB), Blumenau, SC 89030-903 Brazil; 4 Programa de Pós-Graduação Em Ciência E Tecnologia Ambiental, Universidade Do Vale Do Itajaí (UNIVALI), Itajaí, SC 89302-901 Brazil; 5 Universidade Federal Do Paraná (UFPR), Campus Jandaia Do Sul, Curso de Engenharia de Produção, Curso de Engenharia de Produção, Jandaia do Sul, PR 86900-000 Brazil

**Keywords:** Energy recovery, Wastewater sludge, UASB reactor, Greenhouse gas, Environmental sustainability

## Abstract

The objective of this study was to quantitatively assess the energy recovery potential from both biogas and the incineration of dewatered sludge, aiming to meet the energy demand of a small municipal wastewater treatment plant (MWTP) with a treatment capacity below 50 L s^−1^. The energy potential of sludge incineration was characterized based on its gross calorific value, while energy production from biogas generated during the anaerobic digestion of municipal sludge was also evaluated. In addition, the feasibility of using the produced biogas for the thermal drying of dewatered sludge was analyzed. The results indicate that biogas utilization could generate 1462 kWh d^−1^, corresponding to 83.17% of the total electricity consumption of the MWTP. Furthermore, the mass reduction achieved through sludge drying resulted in an annual cost savings of US$ 27,763 related to sludge disposal. The incineration of dried sludge could generate an additional 432.52 kWh d^−1^. Overall, the combined energy recovery strategy could lead to a reduction of approximately 150 t CO_2_eq year^−1^ in greenhouse gas emissions due to methane avoidance at the studied MWTP. These findings suggest that such integrated approaches can encourage investments in MWTPs aimed at achieving energy self-sufficiency while enhancing environmental performance.

**Graphical abstract:**

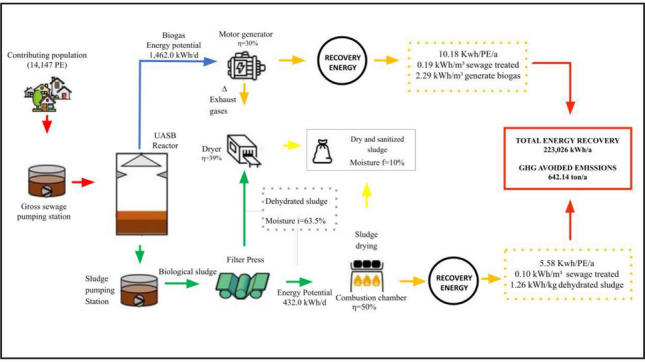

## Introduction

Energy self-sufficiency is increasingly regarded as a critical objective for wastewater treatment plants (WTPs) aiming to comply with rapidly evolving environmental regulatory standards in a sustainable and long-term resilient manner. Upflow anaerobic sludge blanket (UASB) reactors are well established and suitable technologies for wastewater treatment, with widespread application in tropical countries worldwide (Dias et al. 2018; Centeno-Mora et al. 2020). It is considered a low-cost treatment alternative, requiring a relatively small land area for implementation and exhibiting low electricity consumption. However, its operation is associated with the generation of by-products such as sludge and biogas, which require appropriate management (Rosa et al. 2018; Centeno-Mora et al. 2020). The selection of final disposal options for these by-products generally considers locally available alternatives, such as sludge landfilling and biogas flaring (Rosa et al. 2016), since investments in wastewater treatment systems often do not extend to technologies for the valorization of these process by-products. Numerous energy self-sufficient wastewater treatment schemes have been proposed, each tailored to the technical characteristics and constraints of the specific situations analyzed (Longo et al. 2016; Sarpong et al. 2019; Sarpong and Gude 2020; Song et al. 2025). The treatment of municipal wastewater using anaerobic reactors has the potential to shift wastewater treatment plants from energy-consuming facilities to energy-producing systems (Owusu-Agyeman et al. 2021; Ahmad and Senaidi 2023; Shi et al. 2024). The main components of biogas generated in these systems include methane (CH_4_), hydrogen sulfide (H_2_S), nitrogen oxides (N_2_O and NO_2_), and carbon dioxide (CO_2_). Among these constituents, CH_4_ can be directly combusted from UASB reactors or utilized for energy generation in large-scale facilities, resulting in both environmental and economic benefits (Peres et al. 2019). When not recovered, these greenhouse gases (GHGs), particularly CO_2_, CH_4_, and N_2_O, are released into the atmosphere, significantly contributing to climate change (Kiselev et al. 2019; Centeno-Mora et al. 2020). In this context, wastewater treatment plants are widely recognized as significant global emitters of greenhouse gases, ranking among the top ten sources and accounting for approximately 2–3% of total global carbon emissions (Liu et al. 2025). In the face of global challenges such as climate change, the need for greenhouse gas (GHG) emission reduction, and increasing energy demand, energy recovery from wastewater treatment by-products represents a viable strategy for improving energy efficiency in municipal wastewater treatment plants (MWTPs) (Björklund et al. 2001; Ali et al. 2021; Zhang et al. 2024). Ideally, MWTPs should reduce their dependence on grid electricity and move toward energy self-sufficiency in wastewater treatment processes (Sanaye et al. 2022; Ahmad and Senaidi 2023; Blanco et al. 2023; Shi et al. 2024). This can be achieved through the upgrading of biogas to biomethane, which may replace natural gas or be used to generate electricity and thermal energy for drying the sludge produced at MWTPs (Passos et al. 2020). In addition, such approaches offer environmental benefits by avoiding GHG emissions to the atmosphere (Đurđević et al. 2019; Centeno-Mora et al. 2020).

In Brazil and many other tropical countries, the use of biogas for thermal and electrical energy generation remains largely underestimated, despite the widespread implementation of UASB reactors and the substantial quantities of dewatered sludge produced. Moreover, the adoption of energy recovery technologies in these contexts is still incipient (Brasil 2017; Lobato et al. 2018; Comineti et al. 2025). Compared to conventional sludge management practices, energy recovery pathways can represent a transition toward energy self-sufficiency in wastewater treatment plants through the generation of renewable energy rather than mere energy consumption (Lobato et al. 2012; Rosa et al. 2016; Vatachi 2019). Furthermore, sludge combustion reduces solid content, promotes sludge stabilization, eliminates pathogens, and mitigates odor generation, thereby enabling safer final disposal of this solid waste (Di Capua et al. 2020).

To achieve energy self-sufficient treatment systems, the integrated management of process by-products plays a crucial role in minimizing the economic and environmental impacts associated with conventional waste stream management practices (Lobato 2011). Wastewater treatment has evolved toward a new paradigm focused on resource recovery, transforming conventional wastewater treatment plants into resource recovery facilities (Larriba et al. 2020; Comineti et al. 2025). This shift has driven the development of innovative technologies capable of producing high-quality effluents while reducing energy demand and operational costs, thereby supporting the transition of treatment plants into sustainable and economically viable systems (Soares 2020; Romero-Güiza et al. 2022; Centeno-Mora et al. 2020).

Energy recovery through the combustion of methane and dewatered sludge aligns with strategies aimed at meeting the growing energy demands of the sanitation sector (Rosa et al. 2018; Mainardis et al. 2020; Centeno-Mora et al. 2020).

In this context, the objective of this study was to assess the energy recovery potential, process economic balance, and greenhouse gas emission avoidance associated with by-products generated in small MWTP with treatment capacities below 50 L s^−1^, with the goal of achieving energy self-sufficiency. This approach has the potential to significantly contribute to greenhouse gas mitigation and climate protection through energy optimization strategies. To accomplish this objective, a comprehensive dataset was collected and applied to mathematical model equations to evaluate the feasibility of achieving energy self-sufficiency in an MWTP.

## Materials and methods

### Experimental site

The MWTP studied is operated by the Companhia Catarinense de Águas e Saneamento (CASAN) and is located in the Nações district of the municipality of Indaial, in the state of Santa Catarina, southern Brazil. The Nações district is situated within the Itajaí-Açu River Basin, the largest watershed in the state, which drains into the South Atlantic Ocean. This basin is characterized by a dense hydrographic network comprising numerous rivers and streams. Key characteristics of the basin include a population density of 236.5 inhabitants km^−2^, a drainage area of approximately 15,500 km^2^, a main river length of about 70 km, an average discharge of 204.2 m3 s^−1^, and a mixed semidiurnal tidal regime with an average amplitude of 0.8 m.

The MWTP was designed to treat a wastewater flow rate of 55 L s^−1^ and currently operates at an average flow of 23 L s^−1^ (1987 m^3^ d^−1^). The treatment flow diagram comprises preliminary treatment units, including screening, grit removal, and grease traps, followed by sequential anaerobic and aerobic biological treatment units consisting of an upflow anaerobic sludge blanket (UASB) reactor and a submerged aerated biofilter. Sludge dewatering is performed using a filter press (CASAN 2021).

At present, biogas produced in the UASB reactor is flared to prevent methane emissions to the atmosphere, while sludge withdrawn from the bottom of the UASB reactor is conveyed to a booster pumping station prior to dewatering in the filter press. The dewatered sludge is subsequently disposed of in a private landfill in accordance with local environmental regulations.

### Biogas characterization

Volumetric biogas production was quantified using the ProBio 1.0 software, which is based on the mathematical model proposed by Lobato et al. (2012) for estimating biogas generation in UASB reactors. This model applies a mass balance approach that accounts for chemical oxygen demand (COD) conversion pathways and all methane fluxes within the reactor. The software employs a set of equations to determine COD balances, biogas production, and the corresponding energy recovery potential.

Input data required for biogas production modeling in ProBio 1.0 were obtained from November 2023 to November 2024 through the collection and processing of operational data monitored by the utility company responsible for plant operation (CASAN). When specific data were unavailable, default values representing typical operating conditions were adopted based on literature sources to facilitate the calculations. The measured and assumed input parameters used to estimate the biogas energy potential are presented in Table 1.

**Table 1:** Input data for estimating biogas production in ProBio 1.0

Parameters	Unit	Scenario	References
Contributing population (Pop)	hab	14,147	CASAN 2021
Average sewage flow (Qmed)	m^3^·d^−1^	2117.80	CASAN 2021
Tributary COD concentration (COD tributary)	kgCOD·m^−3^	0.515	CASAN 2021
Expected COD removal efficiency (E COD)	%	70.26	CASAN 2021
Concentration of SO_4_^2−^ in the tributary (C SO_4_^2−^)	kg SO_4_^2−^·m^−3^	0.044	CASAN 2021
Sulfate reduction efficiency (E SO_4_^2−^)	%	70	Souza 2010a
Solids production coefficient (Y)	kgSTV·kgCOD_rem−1_	0.15	Andreoli et al. 2014; Lobato et al. 2012
Sludge production coefficient (K _STV-COD_)	kgCOD_sludge_·kgSTV^−1^	1.42	Hoover and Porges 1952
Reactor operating temperature (T)	°C	23.93	CASAN 2021
Loss of CH_4_ dissolved in the treated effluent (pL)	%	20	Paula 2019; Stazi and Tomei 2021
Loss of CH_4_ in the gas phase residual gas (pw)	%	2.5	Souza et al. 2011
Other CH_4_ losses in the gas phase (po)	%	2.5	Souza et al. 2011

Source: adapted from Possetti et al. (2021)

To determine the methane content of the biogas, four samples were collected in duplicate between October and November 2023, with a 15-day interval between sampling events. Biogas samples were collected using plastic syringes equipped with three-way valves at a sampling point located upstream of the gas burner, where a control valve was installed to enable sample collection. The samples were analyzed by gas chromatography using a gas chromatograph equipped with flame ionization and thermal conductivity detectors (GC-FID/TCD; Shimadzu GC-17), with argon employed as the carrier gas. This analytical setup allowed the quantification of CH_4_, CO_2_, O_2_, and N_2_ concentrations in the biogas produced by the UASB reactor.

### Sludge characterization

The production rate of processed sludge was monitored from November 2023 to November 2024 by weighing the dewatered sludge containers prior to their disposal at a landfill. For sludge characterization, samples of dewatered sludge cakes were collected from the filter press between November 2023 and January 2024 and immediately homogenized for subsequent elemental analysis and determination of gross calorific value. Moisture content was determined using a halogen moisture analyzer (ID200, MARTE), following the procedures specified in the manufacturer’s instruction manual, with drying conducted at 105 °C for 60 min.

Volatile solids and ash contents were determined by an external laboratory in accordance with APHA, AWWA, and WEF Standard Methods (2017) . Residual carbon content was estimated indirectly as the difference between total solids and the sum of volatile solids and ash fractions. The elemental composition of the sludge and its gross calorific value were subsequently estimated using empirical correlations based on the measured parameters, as described by the equations presented in Table 2.

**Table 2:** Correlation equations used to estimate the main sludge parameters

Parameters	Equation	Reference
Carbon percentage (%)	C = 0.637(RC) + 0.455(SV)	Parikh et al. 2007
Hydrogen percentage (%)	H = 0.052(RC) + 0.062(SV)	Parikh et al. 2007
Oxygen percentage (%)	O = 0.304(RC) + 0.476(SV)	Parikh et al. 2007
Higher calor. power (MJ·kg^−1^)	PCS = (0.3536 × RC) + (0.1559 × SV)—(0.0078 × AS)	Parikh et al. 2005
Lower calor. power (Kcal·kg^−1^)	PCI = PCS − ((600 × 9.H)/100)	Souza 2010b

*SV* percentage of solid volatile, *RC* residual carbon percentage, *AS* ash percentage

To further investigate the gross calorific value (GCV) of the sludge and to compare the experimentally determined values with those theoretically estimated from correlations based on sludge elemental composition, sludge samples were combusted in a bomb calorimeter to determine the experimental GCV in accordance with the ASTM D240 standard (ASTM 2019).

### Energy generation potential from by-products and energy consumption at the MWTP

The energy recovery potential of the investigated MWTP corresponds to the total energy that could be generated through the combustion of both sludge and biogas produced during the wastewater treatment process. Table 3 presents the variables and corresponding equations used to estimate the energy potential of the MWTP. During the monitoring period, the average annual energy demand of the MWTP was 173,250 kWh year^−1^, with the pumping station accounting for the majority of electricity consumption. The electricity tariff charged by the utility company for operating the MWTP averaged R$ 0.79 kWh^−1^ (equivalent to US$ 0.15 kWh^−1^).

**Table 3:** Equations used to calculate the energy recovery potential of the investigated MWTP

Energy potential of MWTP by-products	PE_total_ = PE_biogas_ + PE_sludge_	PE_total_: total energy potential (MJ·d^−1^)
		PE_biogas_: biogas energy potential (MJ·d^−1^)
		PE_sludge_: sludge energy potential (MJ·d^−1^)
Energy potential of biogas	PE_biogas_ = Q_biogas_ × C_CH4_ × E_CH4_	Q_biogas_: biogas production (Nm^3^·d^−1^)
		C_CH4_: methane concentration in biogas (%)
		E_CH4_: lower calorific value of methane combustion (35.9 MJ·Nm^−3^)
Sludge energy potential	PE_sludge_ = P_sludge_ × PCI	P_sludge_: dehydrated sludge production (kg·d^−1^)
		PCI: lower calorific value of sludge (MJ·kg^−1^)

Source: adapted from Rosa et al. 2016

### Assessment of gaseous emissions and particulate matters from sludge incineration

To determine the gaseous emissions generated during sludge combustion, a 20 g sample of dewatered sludge obtained from the filter press was dried by infrared radiation at 105 °C until constant mass was achieved. The dried samples were then placed in porcelain crucibles and combusted in a muffle furnace (QUIMIS, model Q318M25T) at 530 °C for 20 min. Gaseous and particulate emissions were measured using portable monitoring equipment. Sulfur dioxide (SO_2_), nitrogen dioxide (NO_2_), and carbon monoxide (CO) concentrations were measured using a portable multi-gas detector (VENTIS MX4). Particulate matter with aerodynamic diameters below 2.5 µm (PM_2.5_) and between 2.5 and 10.0 µm (PM_10_), as well as carbon dioxide (CO_2_), was measured using an air quality monitor (TEMTOP, model P1000). Methane emissions avoided through biogas energy recovery were estimated following the methodology recommended by the Intergovernmental Panel on Climate Change (IPCC 2006) for greenhouse gas inventories. The equations and emission factors applied in this assessment are presented in Table 4.

**Table 4:** Equations used to calculate avoided methane emissions

Parcels	Equations	Observations
Emission CH_4_	ECH_4_ = F_E_ × (CODremov − CODsludge) − RCH_4_	ECH_4:_ emission of methane CH_4_ (kgCH_4_·d^−1^) F_E_: emission factor (0.20 kgCH_4_·kgCODremov^−1^) CODremov: daily mass of COD removed (kgCOD·d^−1^) CODsludge: daily mass of COD converted into biomass (kgCODsludge·d^−1^) RCH_4_: CH_4_ removed in energy use (kgCH_4_·d^−1^)
Equation of ideal gases	*P* × *V* = *m* × *R* × (*T* + 273)/MMCH_4_	P: atmospheric pressure (1 atm) *V*: normalized actual production of CH_4_ (m^3^·d^−1^) *m*: mass production of CH_4_ (kgCH_4_·d^−1^) *R*: gas constant (0.08206 atm·L·mol^−1^·K^−1^) *T*: reactor operating temperature (°C) MM CH_4_: molecular mass of CH_4_ (16 g·mol^−1^)

Source: adapted from IPCC (2006)

## Results and discussion

### Biogas characterization

From the treatment and statistical analysis of data collected in the period measured, it was possible to find an average flow of 23.93 L·s^−1^, with a chemical oxygen demand average influent of 515 mg·L^−1^, as shown in Table 5.

**Table 5:** Descriptive statistics of the monitoring data of the studied MWTP

Parameter	Unit	*n*	Mean	Median	Minimum	Maximum
Population	hab	14,147				
Contribution *per capita*	m^3^ ·hab^−1^·d^−1^	12	0.1497	0.1452	0.1009	0.2309
Sewage flow	(m^3^·d^−1^)	12	2.067.60	2.009.76	1.398.48	3.144.96
COD affluent	(mg·L^−1^)	12	515.00	481.00	290.00	802.00
COD UASB effluent	(mg·L^−1^)	12	146.58	129.50	78.00	325.00
COD removal efficiency	%	12	70.26	71.37	49.31	84.95
BOD affluent	(mg·L^−1^)	12	229.74	248.05	128.50	306.15
BOD UASB effluent	(mg·L^−1^)	12	27.55	25.96	16.50	42.81
Sulfate affluent	(mg·L^−1^)	12	44.48	40.08	26.00	80.00
pH UASB	pH at 25°C	14	7.09	7.04	6.62	7.63
Reactor T	°C	14	23.93	23.68	20.85	27.25

As shown in Table 6, the results indicate that the average methane concentration in the biogas was 75.3%, which is a common value for anaerobic reactors treating domestic sewage (Lobato 2011). The concentrations of carbon dioxide and oxygen are also within the typical volumetric composition found in biogas produced in anaerobic reactors treating domestic sewage (Lobato 2011).

**Table 6:** Biogas composition of the studied MWTP

Parameter	Unit	*n*	Mean	Median	Minimum	Maximum	Typical situation by Lobato (2011)
CH_4_	%	4	75.32	75.90	69.03	80.46	60–85
CO_2_	%	4	18.86	18.99	17.77	19.67	5–15
O_2_	%	4	n.d.*	n.d.*	n.d.*	n.d.*	Traces
N_2_	%	4	n.d.*	n.d.*	n.d.*	n.d.*	10–25**

*n.d. not identified

**High fraction of nitrogen in biogas from anaerobic reactors, when it occurs, is due to N_2_ dissolved in domestic sewage

Table 7 reports the biogas flow data for the UASB reactor, obtained after entering the data obtained during the period monitoring to perform the calculations in the ProBio 1.0 computer program.

**Table 7:** Biogas and methane production at the MWTP estimated using ProBio 1.0 software

Parameters	Unit	Result
Standardized production of biogas	Nm^3^·d^−1^	17.205
Standardized methane production	Nm^3^·d^−1^	14.680

The biogas generation rates estimated by the computational model were 0.08 Nm^3^ m^−3^ of treated wastewater and 0.22 Nm^3^ kg^−1^ of COD removed. These results indicate that the MWTP operates within the typical ranges reported in the literature (Lobato 2011), reflecting effective conversion of COD into biogas. Nevertheless, the findings also suggest that there remains potential for increasing biogas production at the plant.

### Sludge characterization

#### Immediate analysis

The proximate composition of the dewatered sludge obtained from the filter press of the UASB reactor at the investigated MWTP is presented in Table 8. The moisture content of the dewatered sludge was 68.2%, which is comparable to the value reported by Rosa et al. (2016) (58.7%). However, the thermal utilization of sewage sludge for energy generation is only feasible when its moisture content is sufficiently low to enable self-sustained combustion. Sewage sludge with moisture contents in the range of 70–80% exhibits low calorific potential and therefore requires additional drying to become an attractive energy source (Wzorek 2012).

**Table 8:** Analytical results for the sludge generated at the investigated MWTP

Statistic	Moisture (%)	Total volatile solids (%)	Ashes (%)	Residual carbon* (%)
Mean (± SD) (*n* = 6)	68.24 (± 0.04)	50.21 (± 0.05)	48.43 (± 0.06)	1.38 (± 0.23)

*The percentage of residual carbon in the samples was obtained by calculating the difference between the total mass and the percentages of ash and solids

As shown in Table 8, the average total volatile solids content of the dewatered sludge was 50.21%. This value is comparable to that reported in the literature for anaerobically treated sludge dewatered by filter press in UASB reactors treating domestic wastewater, which was 42.9% (Rosa et al. 2016).

The sludge exhibited an average ash content of 48.4%, indicating that nearly half of the biomass does not contribute to the combustion process. Rosa et al. (2016) reported an ash content of 53.3% for dewatered sludge from a UASB reactor following filter press dewatering, which is slightly higher than the value observed in the present study. This difference suggests that the sludge analyzed in this work has a higher combustible mass fraction. The final parameter evaluated in the proximate analysis was residual carbon content, with an average value of 1.4%, indicating a low residual carbon fraction in the dewatered sludge. This result is consistent with literature data, which report a residual carbon content of 3.8% for similarly treated sludge from a UASB reactor processing domestic wastewater (Rosa et al. 2016).

#### Elemental analysis

The elemental analysis of the sludge samples revealed average contents of carbon, hydrogen, and oxygen of 23.7%, 3.2%, and 24.3%, respectively. The fractions of carbon and hydrogen positively influence the calorific value of the biomass, as these elements contribute to energy release during combustion, whereas higher oxygen content reduces the fuel’s energy density (Borges et al. 2008; Pokorna et al. 2009). Because elemental composition is directly related to calorific value, an increasing proportion of oxygen and hydrogen relative to carbon generally results in a lower calorific value of the biomass (McKendry 2002). The carbon content of the analyzed samples ranged from 18 to 50%. In a study of sludge from a UASB reactor, Lazzari (2018) reported carbon, hydrogen, and oxygen contents of 28.7%, 4.2%, and 39.4%, respectively, while Rosa et al. (2016) observed values of 19.8% for carbon, 3.6% for hydrogen, and 20.5% for oxygen.

#### Gross calorific value

The gross calorific value (GCV) was estimated using the Parikh correlation, which accounts for the residual carbon, volatile matter, and ash contents of the samples. Based on the analysis of six samples, the average estimated GCV was 7.93 MJ kg^−1^. The experimentally determined GCV was 9.12 ± 1.10 MJ kg^−1^, indicating good agreement between the estimated and measured values. However, the experimentally determined GCV exhibited lower variability compared to the values obtained using the Parikh correlation. Literature values reported by Rosa et al. (2016) and Lazzari (2018), namely, 8.7 ± 1.2 MJ kg^−1^ and 9.75 ± 0.84 MJ kg^−1^, respectively, are close to those obtained in the present study. This similarity can be attributed to the comparable treatment configurations in these studies, which involved domestic wastewater treatment plants equipped with UASB reactors and sludge dewatering systems based on filter presses or drying beds.

For comparative purposes, Table 9 presents higher calorific values reported in the literature for biomasses within the same research scope, namely, sewage sludge.

**Table 9:** Higher calorific values of sewage sludge reported in the literature

Biomass	Gross calorific value (MJ·kg^−1^)	Reference
Sewage sludge–UASB reactor, dehydrated by filter press	8.7 ± 1.2	Rosa et al. 2016
Sewage sludge–UASB reactor, dehydrated in drying bed	9.7 ± 0.8	Lazzari 2018
Anaerobic digester sludge for stabilization of dehydrated solid waste	16.2 ± 0.7	Silva 2011
Sewage sludge from activated sludge system, dehydrated by drying bed	20.1 ± 0.6	Borges et al. 2008
Sewage sludge from activated sludge system, dehydrated	12.7	Mânica 2015
Anaerobic digested sewage sludge	12.8	Andreoli et al. 2014

#### Potential for energy generation from biogas

Based on the volumetric biogas production rate of 17.205 Nm^3^ d^−1^ and an average biogas composition containing 75.32% CH_4_, a gross energy potential of 5269 MJ d^−1^ (equivalent to 1462 kWh d^−1^) was estimated. Accordingly, the net electricity generation from biogas utilization in an internal combustion engine, assuming an electrical efficiency of 39% and a conversion factor of 90%, as proposed by Valente (2015), would be approximately 394 kWh d^−1^, corresponding to about 144,000 kWh year^−1^. This level of energy production represents approximately 83% energy self-sufficiency of the MWTP and could result in annual cost savings of around US$ 22,028.

Previous studies have reported that wastewater treatment plants employing UASB reactors followed by post-treatment processes are, on average, capable of generating sufficient energy to meet at least 59% of their own electricity demand (Santos et al. 2016).

### Energy generation potential from by-products

In addition, the waste heat recovered from the exhaust gases of the internal combustion engine could be utilized for sludge drying and thermal treatment of the sludge generated in the UASB reactor. This approach could reduce the annual mass of sludge disposed of in landfills by up to 63.5%. When considering both electricity generation and the associated reduction in energy procurement costs, as well as savings related to decreased sludge mass, transportation, and disposal expenses, total annual savings of approximately US$ 27,763 could be achieved at the investigated MWTP.

#### Energy generation potential from sludge

With respect to dewatered sludge production over a 1-year period, the average annual generation was estimated at 62,400 kg year^−1^. Based on this value, the potential energy generation from sludge combustion was estimated at approximately 569,088 MJ year^−1^, corresponding to 157.87 MWh year^−1^, considering sludge with a moisture content of 68.4%. This energy potential is associated with the calorific value of the volatile solids fraction of the sludge, estimated at 2.53 kWh kg^−1^. Assuming an electrical conversion efficiency of 50%, as suggested by Santos (2012), the recoverable electrical energy at the investigated MWTP would be approximately 78,936 kWh year^−1^. Considering an electricity tariff of R$ 0.79 kWh^−1^ applied by the utility company, energy recovery through sludge combustion could result in annual cost savings of approximately US$ 12,075.

Regarding gaseous emissions generated during sludge combustion, Table 10 presents the basic air quality measurements. Analysis of the emitted gas composition revealed a high concentration of carbon dioxide relative to carbon monoxide. The low CO concentrations observed suggest efficient combustion of the sludge, with minimal energy losses associated with incomplete combustion, since CO is commonly used as an indicator of combustion efficiency. Additionally, the low concentrations of nitrogen dioxide (NO_2_) and sulfur dioxide (SO_2_) indicate relatively low nitrogen and sulfur contents in the analyzed sludge samples. Similar behavior was reported by Borges et al. (2008), who did not detect these gases during the combustion of domestic sewage sludge samples.

**Table 10:** Gaseous emissions and particulate matter generated during sludge combustion at the MWTP

Parameter	CO (µg·m^−3^)	CO_2_ (µg·m^−3^)	NO_2_ (µg·m^−3^)	SO_2_ (µg·m^−3^)	PM10 (µg·m^−3^)	PM2.5 (µg·m^−3^)
Mean (± SD) (*n* = 5)	29.40 (± 5.08)	1231.80 (± 226.72)	10.0 (± 2.0)	6.0 (± 0.8)	> 999.9	> 999.9

With respect to particulate matter (PM) emissions, high concentrations of both fine particles (PM_2.5_) and coarse particles (PM_10_) were observed, with measured values exceeding the detection limit of the monitoring equipment (> 999.99 µg m^−3^). These concentrations are higher than the limits established by Brazilian environmental regulations for atmospheric PM emissions (CONAMA 2006, 2011, 2018). It should be noted, however, that the combustion process evaluated in this study was not optimized, which may have contributed to the elevated PM levels.

Regarding methane emissions avoided through the implementation of a biogas energy recovery system, it was estimated that 14.68 kg CH_4_ d^−1^ could be avoided at the investigated MWTP. Based on the stoichiometry of methane combustion (CH_4_ + 2O_2_ → CO_2_ + 2H_2_O) and considering that methane has a global warming potential 28 times greater than that of carbon dioxide over a 100-year horizon (IPCC 2014), the reduction in greenhouse gas emissions associated with methane avoidance was estimated at 411.04 kg CO_2_eq d^−1^, corresponding to approximately 150 t year^−1^.

Overall, the quantity and complexity of wastewater entering MWTPs continue to increase, and sustainability challenges extend beyond energy recovery alone. The composition of these wastewaters poses additional concerns related to the presence of pharmaceutical residues, heavy metals, per- and polyfluoroalkyl substances (PFAS), and other emerging contaminants, thereby increasing the complexity and cost of integrated waste management strategies.

## Conclusions

The net energy potentially available from the reuse of by-products for electricity generation at the MWTP in Indaial (Brazil) would be sufficient to supply approximately 83% of the energy demand of the investigated facility. In addition to biogas utilization, the combustion of sludge generated in the UASB reactor after dewatering also demonstrated significant energy potential. Consequently, the total net energy that could be recovered from both biogas and sludge amounts to approximately 610 kWh d^−1^ or 222 MWh year^−1^. These results indicate that both by-products possess relevant energetic characteristics and justify further investment and research focused on energy recovery strategies for MWTPs.

From a climate perspective, the implementation of these energy recovery pathways could avoid approximately 150 t CO_2_eq year^−1^ through methane combustion. The future exploitation of these energy sources could be carried out either individually or in integrated configurations, including cogeneration schemes and hybrid systems combined with renewable sources such as solar energy, thereby enhancing resource management and by-product valorization in the sanitation sector.

Comprehensive assessments of energy performance, economic feasibility, and climate impacts are essential to support decision-making by sanitation service providers. Characterization results such as those obtained in this study are critical to fostering the necessary transformations in management practices, as well as in the design, operation, and maintenance of wastewater treatment facilities. Ultimately, these findings can encourage investments aimed at transitioning MWTPs toward energy self-sufficiency. Future research should focus on detailed economic feasibility analyses of biogas and sludge energy recovery systems in order to further demonstrate the financial and environmental benefits of energetically self-sustainable MWTPs.

## Data Availability

The datasets used and/or analyzed during the current study are available from the corresponding author on reasonable request.
